# Pilot Studies of Estrogen-Related Physical Findings in Infants

**DOI:** 10.1289/ehp.10409

**Published:** 2007-12-12

**Authors:** Judy C. Bernbaum, David M. Umbach, N. Beth Ragan, Jeanne L. Ballard, Janet I. Archer, Holly Schmidt-Davis, Walter J. Rogan

**Affiliations:** 1 Department of Pediatrics, Children’s Hospital of Philadelphia, University of Pennsylvania, Philadelphia, Pennsylvania, USA; 2 Biostatistics Branch and; 3 Epidemiology Branch, National Institute of Environmental Health Sciences, National Institutes of Health, Department of Health and Human Services, Research Triangle Park, North Carolina, USA; 4 Department of Neonatology, Children’s Hospital Medical Center, University of Cincinnati, Cincinnati, Ohio, USA; 5 Survey & Epidemiology Services Division, Social & Scientific Systems, Inc. (formerly Coda, Inc.), Durham, North Carolina, USA; 6 Westat Inc., Durham, North Carolina, USA

**Keywords:** breast bud, estrogen, human milk, infant formula, testis, vaginal maturation index

## Abstract

**Background:**

Soy formula containing estrogenic isoflavones is widely used in the United States. Infants consuming soy formula exclusively have high isoflavone exposures. We wanted to study whether soy formula prolonged the physiologic estrogenization of newborns, but available quantitative descriptions of the natural history of breast and genital development are inadequate for study design.

**Objective:**

We piloted techniques for assessing infants’ responses to the withdrawal from maternal estrogen and gathered data on breast and genital development in infants at different ages.

**Methods:**

We studied 37 boys and 35 girls, from term pregnancies with normal birth weights, who were < 48 hr to 6 months of age, and residents of Philadelphia, Pennsylvania, during 2004–2005. One-third of the children of each sex and age interval were exclusively fed breast milk, soy formula, or cow-milk formula. Our cross-sectional study measured breast adipose tissue, breast buds, and testicular volume; observed breast and genital development; and collected vaginal wall cells and information on vaginal discharge. We assessed reliability of the measures.

**Results:**

Breast tissue was maximal at birth and disappeared in older children, consistent with waning maternal estrogen. Genital development did not change by age. Breast-milk secretion and withdrawal bleeding were unusual. Vaginal wall cells showed maximal estrogen effect at birth and then reverted; girls on soy appeared to show reestrogenization at 6 months.

**Conclusions:**

Examination of infants for plausible effects of estrogens is valid and repeatable. Measurement of breast tissue and characterization of vaginal wall cells could be used to evaluate exposures with estrogen-like effects.

In the United States, 25% of infant formula is based on soy protein (American Academy of Pediatrics Committee on Nutrition 1998). Although soy infant formula has been in use for 60 years in the United States, formula-related illness reports have been largely absent, except for case reports of goiter in the late 1950s (van Wyk et al. 1959), a problem resolved by reformulation (Shepard et al. 1960). More recently, an outbreak of thiamine deficiency in Israeli infants in 2003 was attributed to defective soy formula, manufactured exclusively for the Israeli market (Fattal-Valevski et al. 2005). Soy formula contains estrogenic isoflavones (Setchell et al. 1998). The presence of these compounds is the basis of most health claims made for soy foods, especially for postmenopausal women. Adults rarely exceed 25% of calories from soy (Baird et al. 1995); infants fed soy formula get 100%. Although the soy isoflavones are weak estrogens, soy formula contains so much of them that estimates of daily estrogenic dose per kilogram are equivalent to 0.01 to > 1 hormonal contraceptive pill per day, depending on the potency estimate used (Chen and Rogan 2004; Klein 1998).

Soy formulas do not appear to estrogenize human infants, but such an effect might be overlooked because the term newborn is already responding to the mother’s circulating estrogen. The responses of the fetal breast and genitalia form part of the Ballard score for estimating gestational age (Ballard et al. 1979, 1991). If soy formula were to prolong physiologic estrogenization, it would not be noticed unless it was striking or specifically sought. The U.S. National Toxicology Program Center for the Evaluation of Risks to Human Reproduction Expert Panel recently reviewed the existing human data on the developmental toxicity of soy formula. The panel found the data insufficient to conclude whether soy formula would produce reproductive toxicity. They also noted that the existing human data were “limited by small samples sizes, conflicting results, and historical studies that used soy infant formula of different composition than the formula being sold currently” (Rozman et al. 2006).

In designing a study to test the hypothesis that soy formula prolonged physiologic estrogenization, we found the natural history of estrogen effects *ex utero* not sufficiently well described. We thus undertook a pilot study of breast and genital anatomy, milk secretion, and vaginal cytology over the first 6 months of life, which we report here. The purpose of this study was explicitly to examine methods and to assess which of the characteristics examined might exhibit declines from younger to older children, a pattern consistent with response to the withdrawal of maternal estrogen.

## Patients and Methods

### Recruitment and study design

This physical examination pilot study was conducted at the Children’s Hospital of Philadelphia (CHOP) and affiliated clinics during 2004–2005. The institutional review boards at CHOP, the University of Pennsylvania, and the National Institute of Environmental Health Sciences approved the study.

We recruited volunteer children from the Hospital of the University of Pennsylvania nursery, and CHOP clinics and satellite clinics. Families had to have been consistent in the child’s feeding regimen (Table 1). Eligibility criteria included gestational age 37–41 weeks, birth weight 2,500–4,500 g, palpable testes if male, and no major illness or birth defect. Families were compensated for meal and travel expenses and given coupons for local food stores. Written informed parental consent was obtained for all participants.

We used a mixed cross-sectional design, with up to four visits over time allowed per child. Our design called for 84 total examinations: two boys and two girls in each of seven age intervals and three feeding regimens (Table 1). A group of infants unselected on feeding regimen would consist mostly of those fed cow milk–based formula, especially as they got older. We thus chose to recruit children who had been fed reasonably homogeneous regimens of breast milk, cow milk–based formula, or soy formula, to guard against unexpected variation in our end points related to feeding method and to ensure that our results would apply more broadly.We allowed feeding variations immediately after birth and in older children (Table 1). We deemed nonsoy feeding immediately after birth insignificant. We allowed some flexibility for older children fed solids. Examiners were not blinded to feeding regimen. We assumed that parental report of feeding history was sufficiently accurate, because the primary purpose of the study was to develop and test methods, not to test hypotheses about feeding group differences.

### Physical examination

Our physical examination is adapted from the New Ballard score (Ballard et al. 1979, 1991). The Ballard score measures, among other things, the progressively greater effect of the mother’s circulating estrogen on the fetus. Ballard scores are commonly used in hospital nurseries in the United States to estimate gestational age in the newborn. We used it to measure the involution of the estrogenized term infant’s organs over the first 6 months, assuming that regression would look like progression in reverse. We examined breasts and genitalia, we noted the presence of milk at the nipple and blood in vaginal discharge, and we collected vaginal wall cells. One author (J.L.B.) conducted a 1-day onsite workshop to train staff before the study began. The examiners were two experienced pediatric nurses. Each examiner performed approximately half of the examinations.

The palpability of adipose tissue in the breast was recorded (yes, no). We observed the infants for breast secretions. We assessed the diameter of breast buds, if present, using a custom set of beads. These disc-shaped beads, in 2-mm increments from 4 mm to 22 mm, were strung in an ordered set. The examiner pinched the breast bud between finger and thumb and simultaneously found the closest bead from the string held in the other hand. Each subject’s measurement was the average of triplicate measurements from each breast.

We used a mockup to train examiners and to assess validity of breast bud measurements. Examiners practiced estimating breast bud size using a padded board with various sized beads placed beneath its chamois cover. The examiner palpated it and estimated the size of the hidden bead using a second exposed set. For validation, the two examiners were tested using a random sequence of hidden beads. Validation was conducted in two sessions about 2 months apart; the first used 20 hidden beads and the second used 15. The first session took place before any study exams; the second came after experience with subjects.

Female external genitalia were assigned one of four categories (enlarged minora, majora and minora equally prominent, majora large and minora small, majora cover clitoris and minora). Appearance of the prepuce was classified as prominent or not. Vaginal discharge was characterized by presence or absence and bloody or not. We collected vaginal wall cells with infant recumbent, opening the infant’s legs and using a cotton-tipped swab to rub the introitus for 10 sec. The swab was placed in a vial of Cyto-Rich preservative and sent to CHOP’s contract laboratory to be processed as a Papanicolaou smear. Slides were read by standard methods and a vaginal maturation index calculated as the percent superficial cells plus half of the percent intermediate cells.

Appearance of the testis (retractile, descending, down, pendulous) and the scrotal rugae (rare, few, good, deep) were recorded. We located and noted the position of testes not in the scrotum. We found that commercial Prader (orchidometer) beads (Prader 1966) were too large and insufficiently graduated for newborns; therefore, we commissioned smaller beads in smaller increments (sizes 0.5–3.0 mL in 0.5-mL increments). We measured each testis once and used the average of the two as each subject’s measurement. Prader beads are accepted as valid (Karaman et al. 2005) for peripubertal boys; in infants, Cassorla et al. (1981) reported using beads of the same sizes we used.

### Statistical analyses

Data analysis consisted of descriptive statistics and tests for age trends. We tested trend using the Cochran–Armitage test (Agresti 1990) for binary outcomes, and the Jonckheere–Terpstra test (Hollander and Wolfe 1973) for polytomous outcomes. We used paired *t*-tests to examine left–right differences in paired organs. We examined the trajectory of maturation index as a function of age and the square root of age using cubic-spline regression techniques that accounted for between-subject and within-subject sources of variation. Statistical analysis employed SAS software (SAS Institute Inc., Cary, NC, USA) or StatXact software (Cytel Software Corporation, Cambridge, MA, USA).

## Results

We have data from 88 physical examinations from 72 children, due to minor overrecruitment. Children were allowed up to four examinations over time. One girl had three examinations, seven girls had two examinations each, and 27 girls had a single examination each, for a total of 44 examinations of 35 girls. One boy had four examinations, four boys had two examinations each, and 32 boys had a single examination each, for a total of 44 examinations of 37 boys. Some analyses have smaller sample sizes because some responses were missing. Race/ethnicity of children was reported by the parent; of the 72 children, 4% were Asian or Pacific Islander, 70% were black not of Hispanic origin, 26% were white not of Hispanic origin, and none were of Hispanic origin.

Children were recruited equally among the three feeding regimens and seven age groups (Table 1). Children recruited into the breast-feeding category at older ages (3–6 months) were allowed supplementation with cow’s milk formula; however data on the proportion of children who were “pure” breast-fed versus those who received supplements of cow’s milk were not collected.

Assessment of breast bud diameter using beads appeared valid and repeatable. For the validity of breast bud assessment, one examiner estimated the diameter of the hidden bead correctly in 40 of 45 trials (89%) and was off by at most one bead (2 mm); the other estimated correctly in 35 of 45 trials (78%) and was also off by at most one bead. Accuracy increased with experience; the rates of correct estimation in the second session were 93% and 87%, respectively. We also assessed repeatability through triplicate measurements of each breast. Although necessarily contemporaneous, the triplicate measurements on a single breast never varied (75 breasts with buds present).

**Figure 1:**
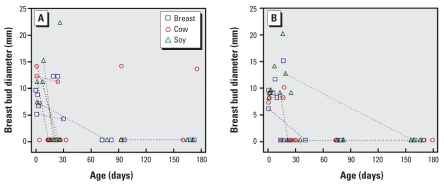
Breast bud diameter (mm) versus age (days) for girls (*A*) and boys (*B*). Line segments connect observations from multiple visits by the same child at different ages.

Average breast bud diameter did not differ between left and right breasts. Maximum difference in an individual was 4 mm in girls and 15 mm in boys. All 12 subjects < 2 days old had palpable buds in both breasts. Breast bud diameter was also maximal within 1 week after birth and was smaller in older children (Figure 1); boys and girls had the same average diameter (Table 2). The proportion of children with palpable buds was smaller in older children (trend *p* < 0.0001 and 0.0003 for boys and girls, respectively); at 1 month or after, only 5 of 20 girls (25%) and 1 of 20 boys (5%) had a palpable breast bud.

Adipose tissue was palpable on at least one breast of every child in every age interval. The proportion palpable on both breasts was smaller in older children, however (trend *p* < 0.0001 and 0.0005 in boys and girls, respectively). Few children had detectable milk secretion at the nipple: 9 of 44 boys (20%) and 5 of 44 girls (11%). All children with detectable breast secretion were older than 2 days but younger than 42 days.

Female genitalia evidenced some change in appearance across age intervals but showed no evidence of postpartum regression. The proportion of girls with detectible vaginal discharge was constant across age intervals; 38 of 44 girls (86%) exhibited vaginal discharge overall, but no blood was observed in any vaginal discharge.

**Figure 2:**
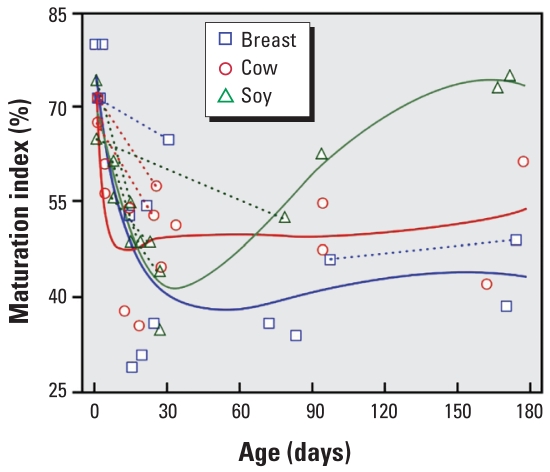
Maturation Index (%) of vaginal wall cells versus age (days). Line segments connect observations from multiple visits by the same child at different ages.

We collected evaluable vaginal wall cell samples from all 44 visits. The maturation index of vaginal wall cells was maximal in newborns, lowest in the 1-month olds, and somewhat higher in the older girls (Figure 2). The curvature was statistically significant (*p* < 0.0001). In addition, feeding regimen appeared to influence the trajectory of maturation index (*p* = 0.07). Differences among feeding regimens appeared minimal among girls younger than about 30 days; among girls older than that, however, girls fed soy formula tended to have higher maturation index.

We saw no consistent differences in scrotal or testicular anatomy at different ages. In every age interval, most of the boys exhibited “good rugae”; overall, only 1 of 44 examinations showed “deep” rugae, 38 showed “good” rugae, whereas the remaining 5 had “few” or “rare” rugae. Most boys (half or more of the boys in every age interval) had both testes “down,” for a total of 31 of 44 boys (70%). Four boys (9%) had both testes retractile; five boys (11%) had both testes pendulous. The remaining examinations were of boys whose testes were in mixed positions: one “descending and down,” and three “down and pendulous.” Mean testis size was constant across age intervals with an upswing in the oldest age interval (6 months) (Table 3). We saw no difference in mean testis volume between left and right sides (*p* = 0.53) with a maximum absolute difference for any individual boy of 0.5 mL.

## Discussion

The techniques of the Ballard scale for physical examination of estrogen responsive tissues in infants are readily taught, and breast bud diameter is validly assessed. Vaginal cytology specimens are easy and atraumatic to collect. All examinations appear precise enough for research protocols.

Not all tissues that show developmental response to estrogen reverted when maternal estrogen was withdrawn at birth. Although breast tissue was maximal in newborns and smaller in older children, external genitalia showed few differences by age. Vaginal bleeding, from withdrawal of maternal estrogen’s trophic effect on the infant’s endometrium, was not observed. Infants synthesize some estrogen, and it may mitigate the effects of withdrawal of maternal estrogen. Vaginal wall cells are responsive to maternal estrogen, appear to lose that effect by 1 month, and then may respond both to estrogen synthesized by the girl and to soy formula. We looked for milk secretion in infants because we thought exogenous estrogen might inhibit prolactin’s promotion of milk synthesis (Guyda and Friesen 1973), but milk secretion was rare. We propose that a longitudinal set of physical examinations of girls aimed at detecting the effects of exogenous estrogen should be performed weekly to fortnightly for the first 1–2 months. Vaginal cytology specimens need to be collected for > 6 months. Milk secretion or vaginal bleeding should be asked about because they have a plausible endocrine basis, but will be infrequent.

Our physical findings, like those of others (Alexander et al. 1992; Donovan et al. 2007), show more variability than Ballard reported (Ballard et al. 1979, 1991) in term infants. Ballard reports labia majora cover the clitoris and minora at term, which we saw in all girls older than 17 days but only in half of the younger girls. Ballard saw deep scrotal rugae at term, but most of our boys had “good” rugae, only one had “deep” rugae.

We measured testicular volume in infants 48 hr to 6 months of age using orchidometer beads that were smaller and in smaller increments (0.5 mL) than the norm. Mean testicular volume (0.7 mL) in our younger age intervals was less than others report, but was similar by 6 months of age (1.04 mL). Average testicular volume from tissue sections in 1-year-olds was 1.1 mL (range 0.3–1.9 mL) in autopsy material from 36 Danish children who died accidentally (Müller and Skakkebæk 1983). Orchidometric assessment in Taiwanese newborns showed mean volume of 1.3 ± 0.3 mL (Chin et al. 1998). Chemes (2001) and Sak et al. (1993) reported infant testicular volumes of approximately 1 mL. In a small but longitudinal study, Cassorla et al. (1981) measured testicular volumes with orchidometer beads of the same sizes as ours monthly in 10 U.S. boys from birth to 6 months of age. Volume increased from birth (1.1 mL) to 2–3 months (2.0 mL), and then declined (1.5 mL at 6 months) (Cassorla et al. 1981). The increase is plausibly related to the reported peak in testosterone at 2 months (Grumbach 2005). Assuming the peak occurs regularly, detecting it may require the smaller variability in longitudinal data, and will require monthly, or more frequent, exams.

Ballard reported that term infants of both sexes had breast buds of 5–10 mm diameter and palpable adipose breast tissue (Ballard et al. 1979, 1991). Among 21 term infants followed by McKiernan and Hull (1981), mean breast bud diameter was 9 mm at birth, 13 mm at 2 weeks, and receded to 10 mm at 1 month, with breast buds in girls larger than in boys. We did not observe an increase in breast bud size postnatally nor a sex differential, and can offer no explanation for these differences. Among 1,126 Danish infants with median age of 3 months, 79% had breast buds, girls more often than boys; buds were larger in girls (5.7 vs. 4.9 mm) and associated with endogenous estradiol levels (Schmidt et al. 2002). McKiernan and Hull (1981) observed that most infants still had palpable adipose breast tissue at 6 months, as did ours, and that most 1-week-old term infants had some milk secretion, but ours did not.

We know of no other studies that report vaginal maturation index from vaginal wall cells in infants, although it can be used both in the diagnosis and the evaluation of treatment for precocious puberty in girls (Comite et al. 1981), and should have a shorter response time than, say, breast size. The components of vaginal maturation index are usually used as a measure of estrogen effects in adult women (Hammond 1977; van der Laak et al. 1999). In a “normal child,” one would expect almost 100% parabasal cells, few intermediate, and 0% superficial cells, for a vaginal maturation index of nearly 0%, whereas a peripubertal girl may have a virtually no parabasal, 90% intermediate, and about 10% superficial for a vaginal maturation index of about 55%. Infant girls’ cells responded as expected to their *in utero* exposure to maternal estrogen (Farage and Maibach 2006); at 1 month, this effect was not apparent. Older girls had higher vaginal maturation index values, which may represent the effect of their own synthesis of estrogen. Older soy-fed girls had somewhat higher vaginal maturation index values than girls of the same age on other diets. This observation, though perhaps a chance occurrence, is plausible and deserving of follow-up. We recommend use of vaginal maturation index in infant girls. A possible parallel measure in boys, called the urocytogram (Preeyasomba and Kenny 1966), exploits the hormonal responsiveness of urethral cells from spun urine. We did not evaluate this end point but now would consider using it.

Our study, although small and primarily cross sectional, suggests choices among responses for further studies. For example, our data suggest that genitalia do not vary much by age and thus will not respond to exogenous estrogen with delayed regression. Breast tissue did appear responsive, as did vaginal wall cells. Withdrawal vaginal bleeding and breast milk are worth noting but are infrequent. This study was a pilot and too small for reliable inference about feeding regimens. Our results indicate that these methods can be used validly and repeatably in infants; they may allow more direct, interpretable investigations of the infant’s response to estrogen-like compounds.

## Figures and Tables

**Table 1:** Nominal age, allowable age ranges, and feeding regimens determining eligibility, 2004–2005.

		Feeding regimens		
Nominal age	Allowable age ranges (days)	Breast milk^a^	Cow milk formula^a^	Soy formula
< 48 hr	age < 2	Breast milk exclusively	Cow milk formula exclusively	Soy formula exclusively
1 week	2 < age ≤10	Breast milk exclusively	Cow milk formula exclusively	Soy formula exclusively
2 weeks	10 ≤age ≤17	Breast milk exclusively	Cow milk formula exclusively	Soy formula exclusively
3 weeks	17 ≤age ≤24	Breast milk exclusively	Cow milk formula exclusively (hospital nursery exception)^b^	Soy formula exclusively (hospital nursery exception)^b^
1 month	24 ≤age ≤42	Breast milk exclusively	Cow milk formula exclusively (hospital nursery exception)^b^	Soy formula exclusively (hospital nursery exception)^b^
3 months	70 ≤age ≤98	Breast milk exclusively or with cow-milk supplementation	Cow milk formula exclusively (hospital nursery exception)^b^	Soy formula^c^ (hospital nursery exception)^b^
6 months	154 < age ≤182	Breast milk exclusively or with cow-milk supplementation	Cow milk formula exclusively (hospital nursery exception)^b^	Soy formula^c^ (hospital nursery exception)^b^

a Restriction: Breast milk and cow milk regimens prohibit any soy foods in baby’s lifetime.

b Hospital nursery exception: Cow milk regimen allowed breast milk, and soy formula regimen allowed breast milk or cow milk while baby was in the hospital nursery immediately after birth. Exclusive feeding of ultimate regimen was required for at least 2 weeks before study examination.

c Soy formula exception: For older children, soy formula regimen must have been fed exclusively and continuously for at least two-thirds of the child’s lifetime, including the 2 weeks before study examination.

**Table 2:** Mean and median breast bud diameter (mm) by sex and nominal age.

	Girls			Boys		
Nominal age	No.	Mean	Median	No.	Mean	Median
< 48 hr	6	9.8	10.3	6	8.0	8.0
1 week	6	9.6	8.5	6	10.2	9.3
2 weeks	6	0.0	0.0	6	7.5	8.0
3 weeks	6	3.8	0.0	6	4.2	2.5
1 month	7	5.1	0.0	7	1.3	0.0
3 months	7	2.0	0.0	6	0.0	0.0
6 months	6	2.3	0.0	7	0.0	0.0

**Table 3:** Mean, minimum, and maximum testis volume (mL) by nominal age.

Nominal age	No.	Mean	Range
< 48 hr	6	0.67	0.5–1.0
1 week	6	0.63	0.5–1.0
2 weeks	6	0.75	0.5–1.0
3 weeks	6	0.71	0.5–1.0
1 month	7	0.75	0.5–1.5
3 months	6	0.67	0.5–1.0
6 months	7	1.04	0.5–1.5
